# The Impact of COMT and Childhood Maltreatment on Suicidal Behaviour in Affective Disorders

**DOI:** 10.1038/s41598-017-19040-z

**Published:** 2018-01-12

**Authors:** Alexandra Bernegger, Klemens Kienesberger, Laura Carlberg, Patrick Swoboda, Birgit Ludwig, Romina Koller, Michelle Inaner, Melanie Zotter, Nestor Kapusta, Martin Aigner, Helmuth Haslacher, Siegfried Kasper, Alexandra Schosser

**Affiliations:** 1 Department of Psychiatry and Psychotherapy, Medical University of Vienna, Währinger Gürtel 18-20, 1090 Vienna, Austria; 2 Department of Psychoanalysis and Psychotherapy, Medical University of Vienna, Währinger Gürtel 18-20, 1090 Vienna, Austria; 3 Karl Landsteiner University for Health Sciences, University Clinic Tulln, Alter Ziegelweg 10, 3430 Tulln, Austria; 4 Department of Laboratory Medicine, Medical University of Vienna, Währinger Gürtel 18-20, 1090 Vienna, Austria; 5 Zentren für Seelische Gesundheit, BBRZ-Med, Schererstrasse 30, 1210 Vienna, Austria

## Abstract

The inconsistent findings on the association between COMT (catecholamine-O-methyl-transferase) and suicidal behaviour gave reason to choose a clear phenotype description of suicidal behaviour and take childhood maltreatment as environmental factor into account. The aim of this candidate-gene-association study was to eliminate heterogeneity within the sample by only recruiting affective disorder patients and find associations between COMT polymorphisms and defined suicidal phenotypes. In a sample of 258 affective disorder patients a detailed clinical assessment (e.g. CTQ, SCAN, HAMD, SBQ-R, VI-SURIAS, LPC) was performed. DNA of peripheral blood samples was genotyped using TaqMan® SNP Genotyping Assays. We observed that the haplotype GAT of rs737865, rs6269, rs4633 is significantly associated with suicide attempt (p = 0.003 [p_corr_ = 0.021]), and that there is a tendency towards self-harming behaviour (p = 0.02 [p_corr_ = 0.08]) and also NSSI (p = 0.03 [p_corr_ = 0.08]), though the p values did not resist multiple testing correction. The same effect we observed with the 4-marker slide window haplotype, GATA of rs737865, rs6269, rs4633, rs4680 (p = 0.009 [p_corr_ = 0.045]). The findings support an association between the COMT gene and suicidal behaviour phenotypes with and without childhood maltreatment as environmental factor.

## Introduction

Suicide has become a major cause of death, with the WHO estimating that suicide accounts for 800.000 of deaths per year throughout the world^1^. Despite efforts of generations of researchers, suicidal behaviour remains disturbingly common and hard to predict^2^.

The major challenge is that affective disorders (AD) and suicidal behaviour (SB) are complex and heterogeneous disorders that are caused by a combination of variations in multiple genes, each exerting a small effect on both disease risk and symptomatical aspects. Furthermore, consistent evidence supports a critical role of environmental factors in modulating or triggering a genetic predisposition^3,4^. Indeed, both early adversity and recent acute/chronic stress have long been recognised to play a pivotal role in both AD and SB^5^. Although SB can occur in different psychopathological conditions (personality disorders, severe anxiety disorders, schizophrenia and other major psychoses), investigators found that affective disorders (both unipolar and bipolar forms) carry the highest risk of SB compared to any other psychiatric and medical illness^6^. Patients with an major depressive disorder have an estimated 6-15% lifetime risk of suicide^7^.

Extensive literature has documented a substantial overlap between AD and SB with regard to familial risk, treatment (e.g., antidepressants and lithium salts are efficacious for major depressive disorder (MDD) and reduce SB risk)^8,9^ and environmental risk factors.

The enzyme catecholamine-O-methyltransferase (COMT) is important for dopamine metabolism and is encoded by the COMT gene, which is located on chromosome 22q11.2. Polymorphisms in this gene affect the metabolism and neurotransmission of dopamine and contribute to the cause of neuropsychiatric disorders like depression^10^.

The most investigated polymorphism (SNP) – rs4680 – coding for a substitution of methionine (Met) for valine (Val) at codon 158, has an effect on the functionality of COMT^11^. The Met allele has one-fourth less enzymatic activity compared to the Val allele. The result of this difference is hypothesised to be that individuals homozygous for the Val allele have a 40% higher enzymatic activity in human dorsolateral prefrontal cortex (PFC), leading to lower synaptic dopamine levels^12^. A study performed by Du *et al*. reported significantly increased COMT mRNA in cortex areas of depressed suicide victims^13^.

The role of dopamine in mood disorders is consistent, since many areas of functioning affected in depression are regulated by this neurotransmitter, for example psychomotor function, motivation, emotion and decision-making^14^.

Studies have shown that the Met-allele patients were more likely to develop depression after exposure to adverse events or having experienced childhood maltreatment^15,16^. Perroud *et al*. investigated that in suicide attempters carrying the Val-allele (protective against MDD) levels of anger were increased in individuals exposed to childhood abuse^17^. These findings may indicate that the protective variant for MD risk may increase SB-related phenotypes (aggressiveness) in combination with early adversity (i.e., environmental risk may increase MD risk in Met-allele carriers, for example, favouring neurotic traits and introversion, while it may increase externalising traits in Val-allele carriers, potentially leading to behaviours such as aggressive-violent self-injury)^18^.

Suicidal behaviour is a very complex phenotype, therefore it should be classified by distinguishing between suicidal thoughts/ideation, self-harm (with or without intent to die), NSSI (non-suicidal self-injury), suicide attempts and completed suicide^19,20^. In a previous study, distinguishing in these suicidal phenotypes, results showed that different types of childhood maltreatment lead to different suicidal phenotypes later in life^21^. Turecki and Brent introduced a similar classification of suicidal phenotypes in order to standardise the nomenclature of suicidal behaviour^22^.

Self-harm includes behaviour directed to oneself with or without intent to die, whereas self-mutilation is defined as intentionally damaging (a) part(s) of the body without conscious wish to die^19,20^. However, mutilators cognitively distinguish their self-mutilation activities from suicide attempts^23^. These phenotypes are of importance, since patients treated in hospital due to injuries resulting from self-harm have a 100 times greater risk of completed suicide than the general population^24^. Therefore the differentiation between suicidal behaviours and a systemic classification to clearly define terms such as non-suicidal self-injury has been found to be important for further research in this field.

Childhood maltreatment, which includes abuse (physical, sexual, and emotional) and neglect (physical and emotional), are a major public health concern, not only for its high prevalence but also because of the consequences and the burden for the people being affected^25^. Researchers showed that childhood maltreatment leads to a range of mental disorders, including depression^26^, and is an important and independent risk factor for suicidal behaviour^27,28^. Further there is evidence that severe early stress and maltreatment induces alterations in brain development, and may have effects on neurogenesis, synaptic overproduction, and myelination during specific periods, which provide the neurobiological basis through which childhood adversity increases the risk of developing psychopathological symptoms^29^.

## Materials and Methods

### Participants, Approval, Accordance, Informed consent

A sample of 258 affective disorder patients were collected at the Department of Psychiatry and Psychotherapy of the Medical University Vienna and the Karl Landsteiner University for Health Sciences, University Clinic Tulln, Austria. The investigation was performed in accordance with the latest version of the Declaration of Helsinki and approval for the study was obtained from the Ethical Committee of the Medical University of Vienna (approval number EK 2013/2013) and the Ethical Committee of the federal state of Lower Austria (approval number GS4-EK4/181/2012). Subjects were recruited according to the following criteria: Inclusion criteria were either ICD-10 or DSM-IV-TR criteria for at least moderate severity of depression or bipolar affective disorder; age 18–65 years (or older if otherwise healthy) and Caucasian ethnicity. Exclusion criteria were lifetime history of schizophrenia, mood incongruent psychotic symptoms, primary substance misuse, primary organic disease, pregnant or breast feeding. The current investigation is part of the FWF funded VieSAD-Study (Vienna Study on Genetics of Suicidal Behaviour in Affective Disorders, KLI°220). It is one of the first data arising from this on-going project and therefore should be considered a pilot study.

After obtaining written informed consent, a detailed clinical assessment through face-to-face interview was performed.

Childhood maltreatment was assessed through the Childhood Trauma Questionnaire Short Form (CTQ-SF)^30^, a retrospective self-report inventory. It compromises 28 items, each rating from 1 (never) to 5 (very often), therefore a high summary score indicates higher levels of childhood trauma. It assesses five types of maltreatment including emotional neglect, emotional abuse, physical neglect, physical abuse and sexual abuse. Sub scores range from 5–25 and thresholds or cut scores have been set for each type of trauma. Cut-offs have been set individually for each CTQ subscale; for emotional neglect 15, emotional abuse 13, physical neglect 10, physical abuse 10, sexual abuse 8. Scores above indicate yes (maltreatment has happened), scores below indicate no (no maltreatment)^31–33^.

To perform a detailed assessment of suicidal behaviour three validated suicidality questionnaires were used: VI-SURIAS (Vienna Suicide Risk Assessment)^34^, SBQ-R (Suicide Behaviour Questionnaire–Revised)^35^, LPC (Lifetime Parasuicidal Count)^36^. For detailed phenotype definition suicidal behaviour was distinguished into subgroups: transient suicidal thoughts, concrete suicidal thoughts, non-suicidal self-injury (NSSI), self-harm, suicide attempt.

### Genotyping

For genetic analyses, 40 ml of venous blood were extracted and DNA isolation was performed using E.Z.N.A Blood DNA mini Kit (Omega bio-tek) according to the manufacturers protocol. To accomplish genotyping, Custom TaqMan®-Genotyping Assay (Applied Biosystems, Rotkreuz, Switzerland) containing specific primers and fluorescence labelled probes for the analysis of the SNPs rs4680, rs4633, rs737865, rs165599, rs9332377, rs6269 was used. For PCR analysis, patients’ DNA was diluted to reach 12ng DNA in 2,25 µl and then 2,5 µl Taqman Universal PCR Master Mix (Applied Biosystems) and 0,25 µl SNP Genotyping Assay Mix (Applied Biosystems) were added. PCR runs were performed on an Applied Biosystems 7900HT Fast-Realtime Thermocycler under the following conditions: 10 min enzyme activation at 95 °C, followed by 40 cycles of 92 °C for 15 s and 60 °C for 1 min. Allelic discrimination by qualitative detection of fluorescence labelled probes was performed using Applied biossystems sds 2.3 software.

### Statistical analysis

Statistical analysis of the data was performed using SPSS 22.0 for Mac OS X (IBM, Armonk USA). All tests were two tailed and the level of significance was set at p < 0,05.

Because childhood trauma (CTQ) total score as well as all sub-scores presented as non-parametric data, Wilcoxon Mann-Whitney U Test was used to assess differences between compared groups in terms of suicidal behaviour.

For comparisons of genotype frequencies within the groups of suicidal phenotypes and gender as categorical variables we performed χ^2^ tests and used the online tool SNPstats (http://bioinfo.iconcolgia.net/SNPstats) to test for Hardy-Weinberg equilibrium. SNPs with a minor allele frequency (MAF) <5% were excluded from further analysis.

To test for gene-environment interaction logistic regression models were developed with subtypes of suicidal behaviour as dependent variable yielding likelihood ratio (L-R) χ^2^ results. Backward stepwise regression was performed with predictor variables being eliminated from the model in an iterative process if significance level was p > 0.1. UNPHASED version 3.1.7 program was applied using three- and four-marker slide windows to analyse for haplotypic association. UNPHASED uses the standard Expectation-Maximisation algorithm in order to estimate haplotypes from genotypes. The rare haplotype frequency threshold was taken as 0.01. UNPHASED uses unconditional logistic regression to perform likelihood ratio tests under a log-linear model of the probability that an allele or haplotype belongs to the case rather than control group. The global null hypothesis is that the odds ratios of all haplotypes are equal between cases and controls. Individual haplotypes were also tested for association by grouping the frequencies of all other haplotypes. All p-values reported in this study were two-tailed. Multiple testing corrections were performed by application of the false discovery rate (FDR)^37^ to both single-marker analyses, it was assumed that 6 independent tests were performed when testing 6 SNPs. Haplotype analyses were corrected for the number of sliding windows used. For risk assessment, logistic regression was performed using backward stepwise procedure to exclude variables not contributing to the model.

### Data availability statement

The manuscript is neither currently under consideration nor published in another journal. There are no restrictions on the availability of materials or information.

### Ethical approval and informed consent

The investigation was performed in accordance with the latest version of the Declaration of Helsinki and approval for the study was obtained from the Ethical Committee of the Medical University of Vienna (approval number EK 2013/2013) and the Ethical Committee of the federal state of Lower Austria (approval number GS4-EK4/181/2012). Every patient signed written informed consent.

## Results

### Descriptive analyses

A total of 255 patients were included (3 patients had to be excluded due to incomplete data), of whom 82.7% were diagnosed with major depressive disorder (MDD) and 17.3% with bipolar disorder (BD). In our sample 35.7% undertook self-harming actions, with or without intent to die, whereas 27.5% attempted suicide at least once in lifetime, and therefore had a concrete wish of ending life, 18.8% of the patients presented with a history of non suicidal self-injury (NSSI), therefore with no intent to die. Transient suicidal thoughts, like weary of life, were reported by 27.50%, and 25.1% had concrete suicidal thoughts. Nineteen point six per cent of the patients never had any suicidal behaviour ever in their life. When we stratified by diagnosis, we found similar suicide attempt rates, with 27% for both MDD and bipolar disorder patients.

According to the predefined cut-off scores of the CTQ subscales, 31.4% experienced emotional abuse, 23.1% had to face physical abuse and 16.5% were sexually abused during childhood. The mean CTQ sexual abuse score of females differs significantly from males, with higher scores in females (p < 0.001). A total of 34.5% of patients were emotionally neglected and 19.6% physically neglected. Patients may have experienced more than one type of childhood maltreatment and therefore scored in more than one subscale of the questionnaire. Forty-five point seven per cent of the patients never experienced any kind of childhood maltreatment.

### SNP on suicidal phenotypes – association analyses

A significant association between the SNP rs737865 and suicide attempts was found in males (p = 0.006 [0.03 after FDR correction]). Two other p-values did not resist multiple testing correction, firstly between rs6269 and concrete suicidal thoughts in the total sample (p = 0.018 [0.9 after FDR correction]), and secondly between rs165599 and concrete suicidal thoughts in males (p = 0.021 [0.1 after FDR correction]). No other associations were found, including the highly investigated rs4680 SNP (Table 1).

**Table 1:** COMT SNPs (single nucleotide polymorphisms) on suicidal phenotypes. Single marker association analyses (SNPs: rs4633, rs4680, rs737865, rs6269, rs165599). Significant p-values in bold. False discovery rate (FDR) p-values after multiple testing correction in brackets. [HW = Hardy-Weinberg equilibrium, COMT = Catechol-O-methyltransferase, NSSI = non-suicidal self-injury, total = total sample].

HW p-value: 0,45	total		female		male	
	χ^2^	p-value	χ^2^	p-value	χ^2^	p-value
rs4633						
transient thought	1.532	0.465	0.041	0.98	2.044	0.36
concrete thought	1.1	0.577	1.881	0.39	1.346	0.51
NSSI	3.785	0.151	3.09	0.213	0.679	0.712
self-harm	2.519	0.284	5.15	0.076	0.256	0.88
suicide attempt	0.929	0.628	0.714	0.7	0.169	0.919
rs4680						
transient thought	1.31	0.519	0.041	0.98	1.575	0.455
concrete thought	1.213	0.545	1.881	0.39	1.777	0.411
NSSI	4.047	0.132	3.09	0.213	1.863	0.394
self-harm	1.88	0.391	5.15	0.076	0.893	0.64
suicide attempt	0.502	0.778	0.714	0.7	0.015	0.993
**HW p-value: 0,46**	**total**		**female**		**male**	
	**χ2**	**p-value**	**χ** ^**2**^	**p-value**	**χ** ^**2**^	**p-value**
rs737865						
transient thought	0.248	0.883	0.357	0.836	0.113	0.945
concrete thought	5.17	0.075	2.687	0.26	2.639	0.267
NSSI	1.422	0.491	2.956	0.228	0.267	0.875
self-harm	0.431	0.806	2.187	0.335	4.72	0.94
suicide attempt	6.08	0.048	1.12	0.57	10.1	**0.006 [0.030]**
**HW p-value: 0,8**	**total**		**female**		**male**	
	**χ** ^**2**^	**p-value**	**χ** ^**2**^	**p-value**	**χ** ^**2**^	**p-value**
rs6269						
transient thought	0.965	0.617	1.034	0.596	2.391	0.302
concrete thought	8.085	0.018 [0.09]	5.549	0.062	2.943	0.23
NSSI	2.635	0.268	1.948	0.378	0.392	0.822
self-harm	1.986	0.37	2.218	0.33	1.599	0.45
suicide attempt	2.383	0.304	0.311	0.856	3.784	0.151
**HW p-value: 0,55**	**total**		**female**		**male**	
	**χ** ^**2**^	**p-value**	**χ** ^**2**^	**p-value**	**χ** ^**2**^	**p-value**
rs165599						
transient thought	2.296	0.317	0.438	0.803	5.625	0.06
concrete thought	3.018	0.221	0.08	0.961	7.722	0.021 [0.1]
non-suicidal self-mutilation	3.256	0.196	0.955	0.622	2.265	0.322
self-harm	1.881	0.39	3.21	0.201	0.118	0.943
suicide attempt	1.027	0.598	0.619	0.734	2.277	0.32

### Risk assessment for SB by logistic regression models: SNPs and CTQ scores

Risk assessment for suicidal behaviour was performed using logistic regression analyses, the cut-off value was ≤0.1. With the suicidal phenotype as the dependent variable Table 2 shows that childhood trauma mainly contributes to the model. The childhood trauma (CTQ total score) effect is constantly significant with all p values ≤ 0.01. The gene effect as the second covariate showed only one promising result, but did not resist multiple testing correction. The SNP rs737865 reveals an association with suicide attempt (p value 0.025 [0.09 after multiple testing correction]). No other associations were found when testing for gene-environment interaction of SNPs and childhood trauma as a combined covariate and suicidal phenotypes as dependent variables.

**Table 2:** Logistic regression - risk assessment for suicidal behaviour (attempted suicide, self-harm behaviour, NSSI = non-suicidal self-injury). Significant p-values in bold, after FDR correction in brackets. (SNP = single nucleotide polymorphism, L-R x2 = likelihood-ratio chi-squared).

SNP	Gene effect		Childhood trauma effect		GxE interaction	
	L-R χ^2^	p-value	L-R χ^2^	p-value	L-R χ^2^	p-value
attempted suicide						
rs4680	0.002	0.961	16.5	**<0.001**	3.46	0.89
rs4633	0.001	0.995	16.48	**<0.001**	4.31	0.9
rs737865	5.05	**0.025 [0.09]**	13.64	**<0.001**	7.21	0.31
rs165599	0.11	0.74	16.35	**<0.001**	7.81	0.62
rs6269	0.297	0.586	16.58	**<0.001**	No valid regression model–omnibus p > 0.05	
self-harm behaviour						
rs4680	1.488	0.22	14.47	**<0.001**	8.44	0.66
rs4633	1.58	0.2	14.26	**<0.001**	9.06	0.6
rs737865	0.12	0.72	14.22	**<0.001**	5.18	0.091
rs165599	1.55	0.68	15.74	**<0.001**	9.664	0.73
rs6269	0.17	0.67	14.08	**<0.001**	9.6	0.055
NSSI						
rs4680	2.1	0.147	9.7	**0.002**	9.07	0.25
rs4633	2.4	0.121	9.46	**0.002**	10.24	0.19
rs737865	1.54	0.21	10.27	**0.001**	9.66	0.13
rs165599	4.17	0.041	10.82	**0.001**	13.13	0.44
rs6269	2.13	0.14	9.44	**0.002**	11.46	0.058

### Haplotype analyses

Haplotypic association analysis of six COMT SNPs revealed significant associations after using 3- and 4-marker slide windows (Table 3). Taking suicide attempt as the dependent variable the haplotypes GAT of rs737865, rs6269, rs4633 (individual p value 0.003 [0.021 after multiple testing correction]) and GATA of rs737865, rs6269, rs4633, rs4680 (individual p value 0.009 [0.045 after multiple testing correction]) were both associated with this suicidal phenotype. For the other suicidal phenotypes, self-harm (GAT p value 0.02 [0.08 after multiple testing correction]) and NSSI (GAT p value 0.03[0.08]), several haplotypes revealed significant p values, but not resisting multiple testing correction.

**Table 3:** Haplotypic association analyses with suicidal phenotypes as dependent variables (suicide attempt, self-harm, NSSI = non-suicidal self-injury). Haplotypes: 3- and 4-marker slide windows of COMT SNPs rs 737865, rs6269, rs4633, rs9332377, rs165599. Significant p-values in bold. False discovery rate (FDR) p-values after multiple testing correction in brackets.

	Haplotypes: 3-marker slide windows				4-marker slide windows		
a) suicide attempt							
rs737865	G				G		
rs6269	A	A			A	A	
rs4633	T	C	C		T	C	C
rs4680		G	G	G	A	G	G
rs9332377			C	C		C	C
rs165599				G			G
Global p	0.031 [0.124]	0.37	0.4	0.62	0.05 [0.22]	0.47	0.54
Individual p	**0.003 [0.021]**	0.34	0.18	0.31	**0.009 [0.045]**	0.23	0.24
Frequency cases/contr	0.025/0.08	0.08/0.05	0.35/0.28	0.11/0.08	0.02/0.07	0.08/0.05	0.11/0.08
b) self-harm							
rs737865	G				G		
rs6269	A	A			A	A	
rs4633	T	T	T		T	T	T
rs4680		A	A	A	A	A	A
rs9332377			T	C		C	C
rs165599				A			A
Global p	0.1	0.25	0.15	0.34	0.18	0.24	0.29
Individual p	0.02 [0.08]	0.11	0.11	0.11	0.052	0.15	0.1
Frequency cases/contr	0.02/0.09	0.48/0.56	0/0.01	0.44/0.52	0.02/0.08	0.48/0.55	0.44/0.52
c) NSSI							
rs737865	G				G		
rs6269	A	A			G	G	
rs4633	T	T	C		C	C	C
rs4680		G	G	C	G	G	G
rs9332377			T	T		T	T
rs165599				G			G
Global p	0.1	0.11	0.02 [0.92]	0.1	0.08	0.03	0.04
Individual p	0.03 [0.08]	0.04 [0.08]	0.03 [0.08]	0.03 [0.08]	0.06	0.03 [0.1]	0.03 [0.1]
Frequency cases/contr	0.03/0.09	0/0.01	0.17/0.08	0.17/0.08	0.27/0.17	0.17/0.08	0.17/0.08

### Gene x environment interaction: Predicted mean suicide risk

Predicted mean suicide risk was calculated by using logistic regression (Table 4). The variables, haplotype GAT of rs737865, rs6269, rs4633 and sexual abuse both contributed to the logistic regression model (SA p < 0.001; GAT p = 0.018), but not the interaction of these two factors (SA*GAT p = 0.114). The most common haplotype in our sample AAT of rs737865, rs6269, rs4633 didn’t show any significance at all.

**Table 4:** Logistic regression; gene-environment-interaction and predicted mean suicide risk. Haplotypes GAT and AAT of rs737865, rs6269, rs4633 and GATA of rs737865, rs6269, rs4633, rs4680. Significant p-values in bold. (GxE = gene-environment, L-R χ^2^ = Likelihood-ratio chi-squared).

Haplotype	Haplotype effect		sexual abuse effect		GxE interaction		model	
	L-R χ^2^	p value	L-R χ^2^	p value	L-R χ^2^	p-value	omnibus χ^2^	p-value
GAT	5.6	0.018	15.6	**<0.001**	2.5	0.114	24.8	**<0.001**
AAT	0.3	0.55	5.2	**0.021**	0.34	0.54	19.4	**<0.001**
GATA	5.6	0.018	15.6	**<0.001**	2.5	0.114	24.8	**<0.001**

## Discussion

The aim of the current study was to investigate a possible association between COMT polymorphisms and defined suicidal behaviour phenotypes in affective disorder patients who have experienced childhood maltreatment.

Because of the fact that the COMT gene encodes for the enzyme Catecholamine-O-Methyltransferase (COMT) and is important for the dopamine metabolism in the prefrontal cortex (PFC)^12^, we hypothesised that the genetic vulnerability due to altered dopamine neurotransmission, the processing of maltreatment during childhood may be impaired and therefore lead to suicidality later in life. As in other studies^38^ we couldn’t find an association between the most common investigated Val158Met SNP (rs4680) and suicidal phenotypes when we tested only for single SNP effects, but in the interaction with childhood emotional abuse. Due to the detailed phenotype description of suicidal behaviour in this study, we found that COMT has a modulating effect when environmental factors, such as childhood maltreatment, are taken into account.

We found an association with suicidal behaviour and the SNP rs737865, with a risk increasing effect for suicide attempt in males (p value = 0.006 [0.030 after multiple testing correction] (Table 1). Gender differences in suicidal behaviour and depression were also suggested in other studies^16,21^. For instance, Aberg *et al*. reported on significant findings within the SNP rs4680, where Swedish men, but not women, with the Met/Met or Met/Val genotypes have a higher risk to develop depression^16^.

The SNP rs737865 doesn’t lead to enzyme-activity changes, but is located in intron 1 region of the gene. This raises the question if this polymorphism leads to a change in transcription rates by altering a transcription factor binding site or adapting alternative splicing processes. Though one study reported that this SNP had no effect on mRNA expression levels, protein immunoreactivity, or enzyme activity^12^ it is recurrently part of different haplotypic analyses. As for instance Schosser *et al*. found an association between single SNPs and haplotypes (e.g. AAT of rs737865, rs6269, rs4633) and suicide risk in treatment resistant depressed patients^39^. In our study, this haplotype did not show an association with suicide risk, but the GAT haplotype of rs737865, rs6269, rs4633 is significantly associated with suicide attempt in our sample (p value = 0.003 [0.021 after multiple testing correction]. The p values for self-harming behaviour and NSSI showed tendencies but did not resist multiple testing correction (Table 3).

Since single SNP effects (especially those which are not leading to alterations in the amino acid code) are mostly inefficient to explain such complex issues like suicidal behaviour, haplotype analysis are a much more promising approach to investigate possible influences and interactions. In the present study we observed that the haplotype GAT of rs737865, rs6269, rs4633 is significantly associated with suicide attempt (p = 0.003 [0.021 after multiple testing correction]), and that there is a tendency towards self-harming behaviour (p = 0.02 [0.08 after multiple testing correction]) and also NSSI (p = 0.03 [0.08 after multiple testing correction]), though the p values did not resist multiple testing correction. Based on previously published findings^21^, in gene x environment haplotypic analyses, we focused on sexual abuse as the strongest environmental factor. Interestingly in the gene x environment regression model to investigate suicide risk, sexual abuse and the haplotype GAT were significantly associated with suicide risk, but not the interaction of these two factors (Table 4). Same effect with the 4-marker slide window haplotype, GATA of rs737865, rs6269, rs4633, rs4680, which is almost the same haplotype than GAT, but with the A allele of rs4680 (p = 0.009 [0.045 after multiple testing correction]). This raises the question if in the 3-marker slide window (GAT of rs737865, rs6269, rs4633), the effect of rs4680 was already included, since rs4633 and rs4680 are located very close to each other on the gene and have almost the same genotypic distribution in our sample. Our most common haplotype in our sample, AAT, did not have an increasing effect on suicidality, such as in the study with treatment resistant depressed patients^39^.

These findings strengthen our hypothesis that the COMT gene might have a modulating effect on suicidal behaviour. Mandelli *et al*. hypothesized that individuals carrying the rs4680 risk variant for MDD (Met-allele = A-allele) may have protective effects toward endophenotypes correlated with SB, like aggressiveness. This possibility of observing the risk-increasing effect of one phenotype and the protective effect for an other phenotype considering a same genetic variant, is an advantage of G x E studies, when studying correlated mental/behaviour disturbance^18^. In our study, the haplotype including the Met-variant (A of rs4680) may have a modulating effect on suicide risk when childhood maltreatment was taken into account (Table 4), but the findings did not show an actual G x E interaction.

Suicidality is associated with personality traits such as novelty seeking and harm avoidance and interestingly the temperament scale novelty seeking (NS) is linked with the dopaminergic system^40^. A recent study performed by Perroud *et al*.^17^ also showed that rs4680 modulates the association between childhood sexual abuse and adulthood anger-trait level. It can be assumed that both the Val- and Met-alleles are involved in the development of psychopathology through different interactions with specific environmental factors.

The hypothesis that COMT affects suicidal behaviour is also strengthened by a study that reports structural and functional impairments within the PFC (prefrontal cortex) in depressed patients, showing hypo-activation, possibly indicating reduced emotion regulation^41^. Furthermore, functional changes due to the SNP rs4680 polymorphism predicted reduced dopamine synthesis in the midbrain and consequently affected the connectivity with the PFC^42^. A recent study showed that in psychotic mood disorder patients, a history of suicidal ideation or suicidal behaviour was associated with PFC-based circuit function in support of cognitive control. The findings of this study also suggest that frontal-based brain dysfunction might directly relate to past worst-point suicidal ideation and/or behaviour, which were key measures of long-term suicide risk^43^. Prior studies showed that the effect of the COMT gene on human cognition is related to the functional polymorphism Val158Met^11^, and increased or decreased enzymatic activity of COMT affects prefrontal cognitive functioning^42^. Goldberg *et al*. found that unique environmental effects of childhood adversity were significantly associated with cognitive performance in subjects homozygous for the Met-allele of the SNP rs4680^44^. Childhood maltreatment itself has strong associations with altered emotional regulation, cognitive functioning and developing suicidal behaviour later in life^21,33,44^. This gives reason that a genetic predisposition might increase the vulnerability for developing such severe behavioural traits and strengthens our findings on COMT and suicidal behaviour.

### Strengths

The strength of this study is that we investigated these different overlapping phenotypes of suicidal behaviour in a homogenous sample of hospitalised patients with diagnosed MDD or BD and excluding patients with primary organic diseases or a history of schizophrenia, to reduce the confounder of comorbidities likely contributing to suicidal behaviour.

### Limitations

Since the CTQ is a self-reported questionnaire; there is a possible underreporting of child abuse experiences by males and to a lower degree also among females. Males often do not report sexual abuse in interviews fearing stigmatisation, vulnerability and loss of masculinity^45^.

Another limitation of the current study is that suicidal phenotypes sometimes have fluent passages and overlaps with other risk behaviour^46^. Most patients showed multiple phenotypes of self-harm, which occur in different time frames and are not always linked to acute episodes of affective disorders. Our detailed phenotype description leads to small sample sizes. Power *et al*. already addressed the limitation of small sample sizes in genetic studies, and that a loss in power due to reduced sample size must be outweighed by the increased power from reduced heterogeneity^47^. Reducing heterogeneity, by only recruiting affective disorder patients and making a detailed suicidal behaviour phenotype description, was a main goal in our study. Aiming to increase sample size, was at a cost of standardized phenotype data in large studies of psychiatric illness^48^. Finding genetic predictors for MDD seem to be more difficult than it is for other psychiatric disorders, due to its complex multifactorial aetiology, therefore in-depth phenotyping should be pursued in tandem, even though it is at a cost of large sample size^47^. Nevertheless replication of significant findings of the current study in larger samples is warranted.

## Conclusion

The findings support an association between the COMT gene and suicidal behaviour phenotypes with and without childhood maltreatment as environmental factor in a sample of patients with affective disorders. Further research with a larger sample size for more statistical power is needed to explore the interactions of the COMT gene.
